# Identification and characterization of the carboxy-terminal region of Sip-1, a novel autoantigen in Behçet's disease

**DOI:** 10.1186/ar1940

**Published:** 2006-04-12

**Authors:** Federica Delunardo, Fabrizio Conti, Paola Margutti, Cristiano Alessandri, Roberta Priori, Alessandra Siracusano, Rachele Riganò, Elisabetta Profumo, Guido Valesini, Maurizio Sorice, Elena Ortona

**Affiliations:** 1 Dipartimento di Malattie Infettive, Parassitarie e Immunomediate, Istituto Superiore di Sanità, Rome, Italy; 2 Dipartimento di Clinica e Terapia Medica Applicata, Cattedra di Reumatologia, Università "La Sapienza", Rome, Italy; 3 Dipartimento di Medicina Sperimentale e Patologia, Università "La Sapienza", Rome, Italy

## Abstract

Given the lack of a serological test specific for Behçet's disease, its diagnosis rests upon clinical criteria. The clinical diagnosis is nevertheless difficult because the disease manifestations vary widely, especially at the onset of disease. The aim of this study was to identify molecules specifically recognized by serum autoantibodies in patients with Behçet's disease and to evaluate their diagnostic value. We screened a cDNA library from human microvascular endothelial cells with serum IgG from two patients with Behçet's disease and isolated a reactive clone specific to the carboxy-terminal subunit of Sip1 (Sip1 C-ter). Using ELISA, we measured IgG, IgM and IgA specific to Sip1 C-ter in patients with various autoimmune diseases characterized by the presence of serum anti-endothelial cell antibodies, such as Behçet's disease, systemic lupus erythematosus, systemic sclerosis and various forms of primary vasculitis, as well as in patients with diseases that share clinical features with Behçet's disease, such as inflammatory bowel disease and uveitis. IgM immunoreactivity to Sip1 C-ter was significantly higher in patients with Behçet's disease and in patients with primary vasculitis than in the other groups of patients and healthy subjects tested (*P* < 10^-4 ^by Mann-Whitney test). ELISA detected IgG specific to Sip1 C-ter in sera from 11/56 (20%) patients with Behçet's disease, IgM in 23/56 (41%) and IgA in 9/54 (17%). No sera from patients with systemic lupus erythematosus, systemic sclerosis, inflammatory bowel disease, uveitis or healthy subjects but 45% of sera from patients with primary vasculitis contained IgM specific to Sip1 C-ter. Serum levels of soluble E-selectin, a marker of endothelial activation and inflammation, correlated with levels of serum IgM anti Sip-1 C-ter in patients with Behçet's disease (r = 0.36, *P* = 0.023). In conclusion, Sip1 C-ter is a novel autoantigen in Behçet's disease. IgM specific to Sip1 C-ter might be useful in clinical practice as an immunological marker of endothelial dysfunction in vasculitis.

## Introduction

Behçet's disease (BD) is a systemic form of primary vasculitis characterized by recurrent oral and genital ulcers and ocular inflammation and with frequent involvement of the joints, central nervous system and gastrointestinal tract. Its aetiology is unknown. The most favoured pathogenetic mechanism is a genetic susceptibility associated with HLA-B gene polymorphisms. Other evidence indicates a pathogenic role for environmental factors, including infectious agents or autoimmune mechanisms [1]. Supporting an immune origin, serum from patients with BD has been found to contain autoantibodies directed against several antigens, among them autoantibodies against the endothelium [2-10]. Because the low specificity and immunoreactivity of these autoantibodies prevents their use in diagnosis, no serological test specific for BD is yet available. The diagnosis is, therefore, based on clinical criteria. The clinical diagnosis of BD is nevertheless difficult because the signs and symptoms vary widely, especially at the onset of disease.

Our primary aim in this study was to seek and characterize endothelial autoantigens specifically recognized by serum autoantibodies in patients with BD that might be a useful tool in the diagnosis of BD. Because vasculitis in patients with BD mainly involves capillaries and small vessels, and because microvascular endothelial cells differ from vein or artery endothelial cells in phenotype [11,12], we used a human microvascular endothelial cell (HMVEC) cDNA expression library to identify target antigens. By screening the library with sera from two patients with BD we identified a strongly reactive clone encoding the carboxy-terminal subunit of the splicing factor Sip1 (Sip1 C-ter). We then used ELISA to measure IgG, IgM and IgA specific to Sip1 C-ter in patients with distinct autoimmune diseases characterized by the presence of serum anti-endothelial cell antibodies such as BD, systemic lupus erythematosus (SLE), systemic sclerosis (SSc), various forms of primary vasculitis as well as in patients with diseases that share clinical features with BD, such as inflammatory bowel disease and uveitis. Finally, we evaluated the correlation of serum antibodies specific to Sip1 C-ter with soluble E-selectin, an established marker of endothelial dysfunction.

## Materials and methods

### Patients

Fifty-six unselected out-patients with BD (17 women, 39 men; mean age 37.7 years, range 14 to 58 years; mean disease duration 8.1 years, range 0 to 24 years) attending the Rheumatology Division of the University of Rome "La Sapienza" were enrolled in the study. All patients fulfilled the diagnostic criteria of the International Study Group for BD [13]. Informed consent was obtained from each patient and the local ethics committee approved the study. Glucocorticoids were used in 46.1% of patients with BD, immunosuppressive drugs (cyclosporine A, methotrexate, azathioprine, chlorambucil) in 56.4%, infliximab in 5.1%, interferon α in 5.1%, and 10.2% of the patients with BD were not treated. Patients who had two of the seven findings (oral and genital ulcerations, skin lesions, eye involvement, positive pathergy test, thrombophlebitis and arthritis), or multiple erythema nodosum with severe inflammation and with both elevated erythrocyte sedimentation rate and positive C-reactive protein were assumed to have active disease. According to these criteria, 42% of patients had active disease. The frequency of the HLAB51 allele was 79%. As control groups, we also enrolled 32 consecutive patients with SLE diagnosed in accordance with the American College of Rheumatology revised criteria for the classification of SLE [14], 24 consecutive patients with SSc diagnosed in accordance with the criteria of the American Rheumatism Association [15], 20 patients with primary vasculitis (9 patients with Wegener's granulomatosis, 4 with Churg-Strauss syndrome, 2 with Takayasu's Arteritis, 2 with Horton disease, 2 with microscopic poly-angiitis, 1 with panarteritis nodosa), 33 patients with inflammatory bowel disease (IBD), 17 patients with uveitis (12 with idiopathic diffused uveitis, 5 with idiopathic anterior uveitis) and 40 healthy subjects, matched for sex and age.

### Immunoscreening of the cDNA expression library

A commercially available HMVEC cDNA library (Stratagene, Cambridge, UK) was screened with a pool of sera from 2 of the 56 patients with BD, essentially as previously described [16]. The serum was diluted 1:100 in PBS containing 1% milk, 0.1% Tween-20 and 0.02% sodium azide. Positive plaques were re-screened with the same pool of sera to obtain the clonality. Immunoreactive cloned phage was recovered as pBluescript by single-stranded rescue using the helper phage (Stratagene) according to the manufacturer's instructions and used to transform SolR XL1 cells. The nucleotide sequence of the cloned cDNA insertion was sequenced with automated sequencer ABI Prism 310 collection (Applied Biosystems, Foster City, CA, USA) and sequences were then compared with the GenBank sequence database using the Blast program.

### Expression and purification of the recombinant antigen

The selected cDNA clone was sub-cloned into the *Bam*HI/*Hin*dIII restriction site of the QIA express vector, pQE30. The fusion protein was expressed in *Escherichia coli *SG130009 cells, purified by affinity of NI-NTA resin for the six-histidine tail and eluted under denaturing conditions according to the manufacturer's instruction (Qiagen, GmbH, Hilden, Germany).

### SDS-PAGE and immunoblotting

After 12% SDS-PAGE under reducing conditions, immunoblotting was performed as previously described [17]. In brief, the antigen was loaded at concentrations of 3 μg/lane and was revealed by human sera diluted 1:100 and by a monoclonal antibody to six-histidine tail (Qiagen). Peroxidase-conjugated goat anti-human and anti-mouse IgG sera (Biorad, Richmond, CA, USA) were used as second antibodies. Strips were developed with 3'-3' diaminobenzidine (Sigma-Aldrich, St Louis, MO, USA).

### Purification of specific autoantibodies from patients' sera

Antigen (50 μg) was spotted onto a nitrocellulose filter and incubated with a patient's serum that was positive in immunoblotting. After washing with PBS-Tween the antibodies were eluted with glycine 100 mM, pH 2.5, and mixed for 10 minutes. The eluted antibodies were immediately neutralized with TRIS-HCl 1 M, pH 8.

### Indirect immunofluorescence assay

An indirect immunofluorescence assay was developed on permeabilized EAhy-926 endothelial cells, as previously described [18]. Cells were incubated with purified human antibodies (0.1 μg/μl) in PBS containing 1% bovine serum albumin. Fluorescein isothiocyanate-conjugated anti-human IgG (Sigma) was added and fluorescence was analysed with an Olympus U RFL microscope (Olympus, Hamburg, Germany). Anti-nuclear antibodies were detected using indirect immunofluorescence with Hep2 cells according to the manufacturer's instructions (Radim Diagnostic, Rome, Italy). Titers of more than 1:80 were considered positive.

### ELISA

ELISA was developed essentially as previously described [18]. In brief, polystyrene plates (Dynex, Berlin, Germany) were coated with the antigen (0.1 μg/well) in 0.05 μM NaHCO_3 _buffer, pH 9.5, and incubated overnight at 4°C. Plates were blocked with 100 μl/well of PBS-Tween containing 3% milk, for 1 hour at room temperature. Human sera were diluted in PBS-Tween and 1% milk (1:100 for total IgG and 1:50 for IgM and IgA), 100 μl per well. Peroxidase conjugates goat anti-human IgG (Biorad, Richmond, CA, USA), anti-human IgA (Sigma) and anti-human IgM (ICN Biomedicals, Costa Mesa, CA, USA) were diluted in PBS-Tween containing 1% milk (1:3,000, 1:3,000 and 1:500 respectively) and incubated 1 hour at room temperature. *O*-phenylenediamine dihydrochloride (Sigma) was used as a substrate and optical density (OD) was measured at 490 nm. Means + 2 standard deviations of the OD reading of the healthy controls were considered as cut-off level for positive reactions. All assays were performed in quadruplicate. Data were presented as the mean OD corrected for background (wells without coated antigen). The results of unknown samples on the plate were accepted if internal controls (two serum samples, one positive and one negative) had an absorbance reading within mean ± 10% of previous readings. To inhibit specific IgG, IgM and IgA, the sera from two patients with BD were incubated overnight at room temperature with 10 μg/ml of Sip1 C-ter according to the method reported by Huang and colleagues [19]. As a negative control, the sera was pre-incubated with 40 μg/ml of bovine serum albumin.

Soluble E-selectin was detected using a sandwich ELISA kit (R&D Systems, Minneapolis, MN, USA). ELISA was performed in accordance with the manufacturer's instructions.

### Statistical analysis

Chi-square analysis was used to evaluate differences between percentages; Kruskal Wallis non-parametric ANOVA test and the Mann-Whitney unpaired test were used to compare quantitative variables. *P *values less than 0.05 were considered to indicate statistical significance. Pearson correlation (r correlation coefficient) and linear regression analysis were used to determine if the levels of soluble serum E-selectin correlated with the levels of anti-Sip1 C-ter antibodies in patients with BD

## Results

### Immunoscreening of the HMVEC expression library

**Figure 1:**
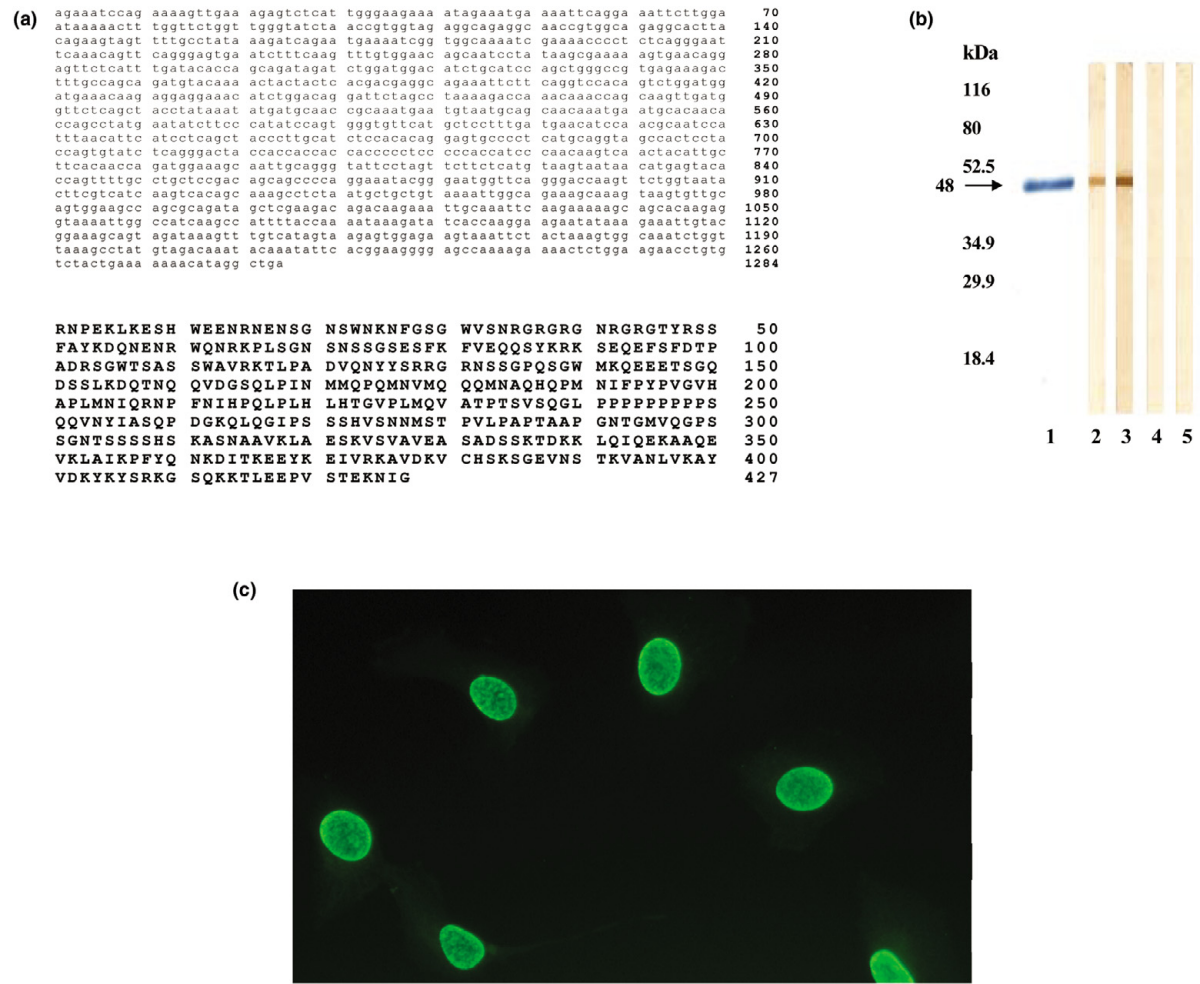
Nucleotide, amino acid sequence and immunochemical characterization of the carboxy-terminal region of Sip1. **(a) **The nucleotide sequence containing 1,281 base-pairs of the cloned cDNA insertion was sequenced with an automated sequencer ABI Prism 310 Collection. The amino acid sequence predicted from the nucleotide sequence is 109 residues long. (Sip1 C-ter; GenBank accession number for Sip1 cDNA is AF030234) **(b) **The molecular size and the purity of the expressed protein was confirmed by 12% SDS-PAGE stained by Coomassie blue (lane 1) and the patients' serum immunoreactivity was analyzed by immunoblotting. Lane 2, the monoclonal antibody anti-histidine tail; lane 3, serum pool from the two patients with BD used in screening the library; lane 4, representative serum from a healthy subject; lane 5, control lane without serum. **(c) **Immunofluorescence analysis of Sip1 localization in EAhy-926 endothelial cells. The human IgG antibodies purified from patient's serum specific to Sip1 C-ter were used to analyse the cellular distribution of Sip1.

Immunoscreening of the HMVEC expression library with IgG from the serum pool of two patients with BD identified one strongly reactive clone. The amino acid sequence of the clone, predicted from the 1,281 base-pair open reading frame of this clone, is 427 residues long and has 100% identity with the carboxy-terminal subunit of the splicing factor Sip1 (Figure 1a). The expected molecular size of 48 kDa, the purity and the immunoreactivity of the expressed protein were confirmed by 12% SDS-PAGE and immunoblotting (Figure 1b). The nuclear localization of Sip1 in endothelial cells was observed in immunofluorescence with patients' antibodies purified from recombinant antigen (Figure 1c).

### ELISA for IgG, IgM and IgA specific to Sip1 C-ter

**Figure 2:**
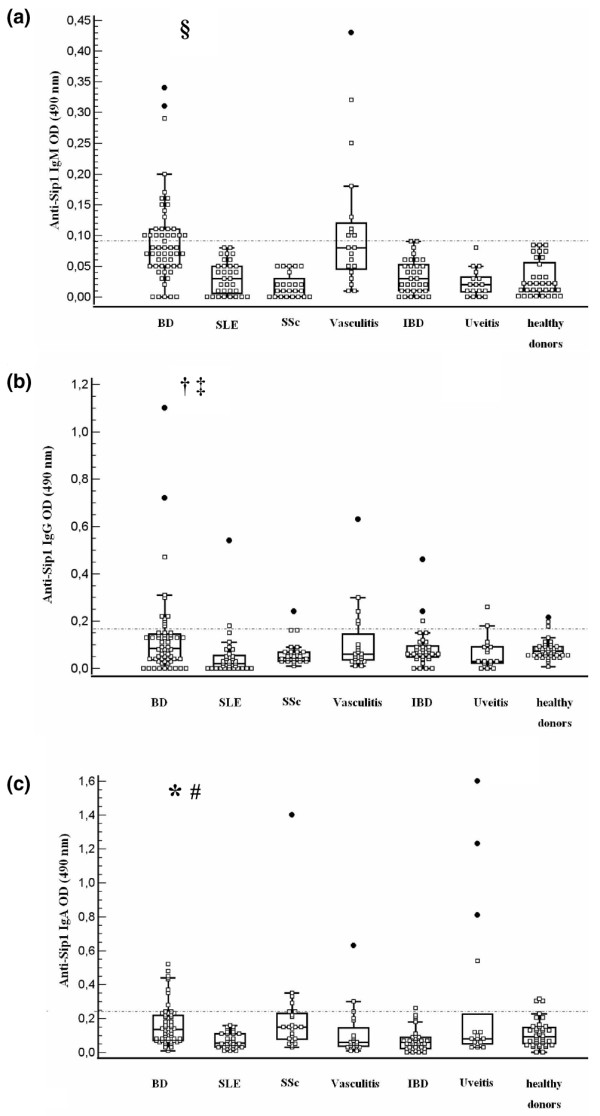
Anti-Sip1 C-ter antibodies in patients and healthy subjects. Anti-carboxy-terminal subunit of Sip1 (anti-Sip1 C-ter) antibodies in patients with Behçet's disease (BD), systemic lupus erythematosus (SLE), systemic sclerosis (SSc), vasculitis, inflammatory bowel disease (IBD), uveitis and from healthy donors. Median, quartiles, range, and possibly extreme values are indicated. The broken line represents the cutoff (mean + 2 standard deviations for the healthy controls). Outliers are represented as solid circles. **(a) **Box-whisker plot of anti-Sip1 C-ter IgM (^§^*P *< 10^-4 ^for BD versus SLE, BD versus SSc, BD versus IBD, BD versus uveitis, BD versus healthy donors). **(b) **Box-whisker plot of anti-Sip1 C-ter IgG (^†^*P *= 0.001 for BD versus SLE; ^‡^*P *= 0.033 for BD versus vasculitis). **(c) **Box-whisker plot of anti-Sip1 C-ter IgA (*P < 10^-4 ^for BD versus SLE, BD versus healthy donors, BD versus IBD; ^#^*P *= 0.012 for BD versus vasculitis). OD, optical density.

Serum IgM, IgG and IgA immunoreactivity to Sip1 C-ter differed significantly between the groups examined (*P *< 10^-4 ^by Kruskal-Wallis test). Serum IgM immunoreactivity to Sip1 C-ter was significantly higher in patients with BD and in patients with other primary vasculitis than in those with SLE, SSc, IBD, uveitis and healthy subjects (*P *< 10^-4 ^by Mann-Whitney) (Figure 2a). Serum IgG immunoreactivity to Sip1 C-ter was higher in patients with BD than in the other groups tested, but the difference was significant only versus patients with SLE and with vasculitis (*P *= 0.001 and *P *= 0.033, respectively, by Mann-Whitney) (Figure 2b). Serum IgA immunoreactivity was significantly higher in patients with BD than in those with SLE, with the other forms of primary vasculitis, with IBD and in healthy subjects (*P *< 10^-4 ^for BD versus SLE, BD versus healthy subjects, BD versus IBD; *P *= 0.012 for BD versus vasculitis by Mann-Whitney) (Figure 2c). The pre-absorption of sera from two patients with BD with Sip1 C-ter itself completely inhibited the antibody reactivity, confirming the specificity of ELISA (data not shown).

ELISA detected IgG specific to Sip1 C-ter in 11/56 (20%) patients with BD, IgM in 23/56 (41%) and IgA in 9/54 (17%). We found IgM specific to Sip1 C-ter in sera from 9/20 (45%) patients with primary vasculitis but in no sera from patients with SLE, SSc, IBD, uveitis or healthy subjects (Table 1).

In patients with BD, we found no significant association between detectable autoantibodies to Sip1 C-ter and clinical manifestations, in particular vascular manifestations (venous and arterial thrombosis, cutaneous or visceral vasculitis) (Table 2). We found no significant association between anti-Sip1 C-ter antibodies and disease activity, therapeutic regimen or HLA B51 expression.

Immunofluorescence analysis detected anti-nuclear antibodies in 7 of the 56 patients with BD (12.5%) at low titer (from 1:80 to 1:160). Five of these seven positive patients had serum antibodies specific to Sip1 C-ter (two patients had serum anti-Sip1 C-ter IgM, two anti-Sip1 C-ter IgM and IgG and one anti-Sip1 C-ter IgM and IgA).

**Figure 3:**
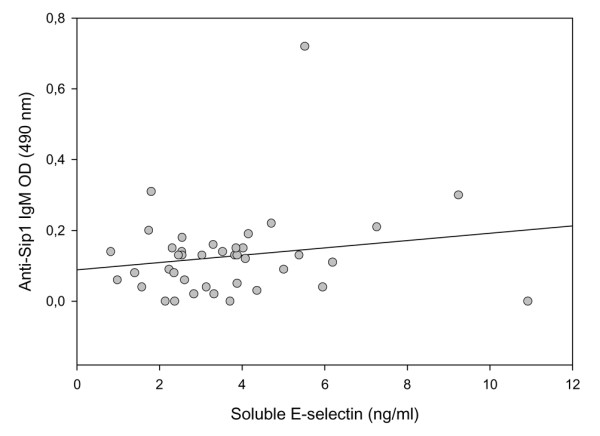
Correlation and linear regression of soluble E-selectin and anti-Sip1 C-ter IgM.l. Correlation and linear regression of soluble E-Selectin (ng/ml) and serum IgM specific to the carboxy-terminal subunit of Sip1 (optical density (OD) 490 nm) in patients with Behçet's disease, correlation coefficient r = 0.36, *P *= 0.023.

In patients with BD, we detected a significant positive correlation between serum IgM specific to Sip1 C-ter and soluble E-selectin serum levels (r = 0.36, *P *= 0.023; Figure 3), whereas we found no significant correlation between anti-Sip1 IgG and IgA and soluble E-selectin.

## Discussion

In this study, by screening an HMVEC cDNA expression library to identify target antigens, we identified a strongly reactive clone encoding the carboxy-terminal subunit of the splicing factor Sip1 (Sip1 C-ter). The carboxy-terminal region of Sip1 – a novel endothelial autoantigen recognized by serum autoantibodies in patients with BD – may be a marker of endothelial dysfunction in vascular autoimmune diseases.

To our knowledge, this is the first report describing an immune response against the protein Sip1. Sip1 is a nuclear splicing factor containing an arginine/serine-rich domain and a RNA-binding motif that may play a role in linking the processes of transcription and pre-mRNA splicing [20]. How Sip1 might become an autoantigen exposed to the immune system and whether this process involves apoptosis need further investigations.

Apoptosis of endothelial cells may be initially induced by inflammation or oxidative stress caused by intrinsic or extrinsic factors. In this environment, mature dendritic cells would process and present Sip1, among other intracellular antigens, to autoreactive lymphocytes, thereby triggering the production of autoantibodies. Antibodies specific to Sip1 could in turn induce additional cellular damage by activating complement or through their cytotoxic properties, penetrating living cells. They might also merely reflect an immune response against antigens released from damaged endothelium. Another possible explanation for the immune response to Sip1 is molecular mimicry. Again, further investigations will clarify the possible cross-reaction with molecules from microorganisms associated with BD and Sip1. Using a molecular strategy to identify autoantigens in BD, Lu and colleagues [4] immunoscreened a T24 cDNA expression library and identified kinectin as a BD autoantigen. Presumably our study and that of Lu and colleagues identified different molecular targets because the cDNA libraries and patient populations differed.

In this study, we used ELISA to analyse Sip1 C-ter immunoreactivity to IgG, IgA and IgM, three immunoglobulin classes potentially involved in the pathogenesis of BD. Kruskal-Wallis test showed that the immunoreactivity of IgM, IgG and IgA specific to Sip1 C-ter varied significantly among various groups of patients analysed. The precise significance of the isotypes of anti-Sip1 antibodies and their potentially independent clinical role remains unclear.

Another important question to clarify is whether distinct autoantibody isotypes recognize different Sip1 epitopes. IgM specific to Sip1 C-ter achieved the highest prevalence and the highest specificity in patients with BD and with vasculitis. Autoimmune diseases can be associated with elevated levels of IgM autoantibodies that may have a pathogenic effect [21]. In particular, IgM antibodies might have an important role in the pathogenesis of BD and high IgM deposition has been found in papulopustular lesions, the most common type of cutaneous lesions in BD and in the vessels of the lesional skin [22].

In patients with BD, anti-endothelium IgM is more frequent than anti-endothelium IgG and is associated with vasculitis [23]. In a recent study using proteomic technology to identify a protein of human dermal microvascular endothelial cells that reacts with anti-endothelial cell antibodies of patients with BD, Lee and colleagues [2] identified α-enolase, another ubiquitous protein, as an autoantigen recognized by serum IgM from patients with BD, thus confirming the importance of anti-endothelium IgM in BD.

The target antigens, Sip1 in our study and α-enolase in the study by Lee and colleagues, were recognized only by IgM of patients with BD and patients with other forms of primary vasculitis, whereas no serum from patients with other autoimmune diseases or from healthy subjects had specific IgM with an OD reading in ELISA higher than the cutoff level. The presence of IgM specific to Sip1 only in the sera from patients with primary vascular disease as well as the positive correlation of serum IgM specific to Sip1 C-ter with the serum levels of soluble E-selectin, a typical marker of endothelial activation and inflammation, suggests the potential role of these antibodies as immunological markers of endothelial dysfunction [24].

The role of IgA specific to Sip1 C-ter in patients with various diseases needs further investigation in a larger sample of patients. Because IgA is a poor activator of complement, this class of immunoglobulins may have a protective function inhibiting complement activation by blocking the binding of IgG or IgM antibodies [25]. Although Sip1 is a nuclear protein, in patients with BD we found no association between serum anti-Sip1 antibodies and anti-nuclear antibodies, probably because we used different techniques to reveal the two antibodies. Further studies are in progress to clarify the effective role of anti-Sip 1 antibodies *in vivo *and to provide new insights into their potential pathogenicity.

## Conclusion

One way of improving the diagnosis of BD is to characterize new autoantigens for use in immunodiagnostic tests. In this study, we identified Sip1 C-ter as a novel autoantigen in BD. Anti-Sip1 C-ter IgM should be useful as a marker of endothelial dysfunction in vasculitis. This recombinant antigen might also provide new insights into the role of specific autoantibodies in the autoimmune mechanisms underlying the pathogenesis of BD.

## Abbreviations

BD = Behçet's disease; ELISA = enzyme-linked immunosorbent assay; HMVEC = human microvascular endothelial cells; IBD = inflammatory bowel disease; OD = optical density; PBS = phosphate-buffered saline; Sip1 C-ter = carboxy-terminal subunit of Sip1; SLE = systemic lupus erythematosus; SSc = systemic sclerosis

## Competing interests

EO, PM and FD are applying for a patent of Istituto Superiore di Sanità relating to content of the manuscript.

## Authors' contributions

FD screened the library, conducted the ELISA experiments and participated in the design of the study and analysis of the data. FC participated in the design of the study and in the analysis of data and helped to draft the manuscript. PM cloned and sequenced cDNA, purified the recombinant protein and helped to interpret the data. CA participated in the design and revision of the study and performed the statistical analysis. RP participated in the design and revision of the study. AS participated in the analysis and interpretation of data and helped to draft the manuscript. RR participated in the design of the study and in the revision of the manuscript. EP participated in analysis of data. GV participated in the design of the study and in the revision of the manuscript. MS conducted the experiments on endothelial cell lines, participated in the design of the study and helped to draft the manuscript. EO conceived the study, participated in its design and coordination and drafted the manuscript. All authors read and approved the final manuscript.

## Figures and Tables

**Table 1:** Distribution of anti-Sip1 C-ter antibodies in sera from patients and from healthy donors

Serum samples	Number of total samples	Anti-Sip1 C-ter antibody response, number of positive samples (%)		
		IgG	IgM	IgA

Behçet's disease	56	11 (20)	23 (41)	9 (17)
Systemic lupus erythematosus	32	2 (6)	0 (0)	0 (0)
Systemic sclerosis	24	1 (4)	0 (0)	4 (17)
Primary vasculitis	20	1 (5)^a^	9 (45)^b^	3 (15)^c^
Inflammatory bowel disease	32	1 (3)	0 (0)	1 (3)
Uveitis	17	2 (11)	0 (0)	4 (23)
Healthy donors	40	2 (5)	0 (0)	3 (7)

Sip1 C-ter, carboxy-terminal subunit of Sip1.

^a ^One patient with microscopic poly-angiitis; ^b ^four patients with Wegener granulomatosis, two with Takayasu's arteritis, two with microscopic poly-angiitis and one with Horton disease; ^c ^one patient with Wegener granulomatosis, one with Churg-Strauss syndrome and one with Takayasu's Arteritis

**Table 2:** Clinical manifestations of patients with Behçhet's disease in relation to anti-Sip1 C-ter autoantibodies

Clinical manifestation of the 56 patients with Behçet's disease	Number of total samples (%)	Anti-Sip1 C-ter antibody response, number of positive samples (%)		
		IgG	IgM	IgA

Skin lesions	36 (64)	7 (19)	14 (39)	7 (19)
Ocular inflammation	38 (68)	8 (21)	16 (42)	5 (13)
Genital aphtosis	27 (48)	5 (18)	8 (29)	5 (18)
Arthritis	10 (18)	1 (10)	6 (60)	2 (20)
Vascular manifestations	10 (18)	4 (40)	6 (60)	4 (40)
Neurological manifestations	6 (11)	0	1 (1.6)	0

Sip1 C-ter, carboxy-terminal subunit of Sip1.
